# Supplementary material for: Integrated analysis of the aqueous humor microbiome and lens capsule transcriptome in high myopia cataract: a pilot study
Source: Front Med (Lausanne). 2026 Jun 16;13:1845205. doi: 10.3389/fmed.2026.1845205 (PMC13314463; doi:10.3389/fmed.2026.1845205)
Supplement: Supplementary file 3 [file Data_Sheet_1.zip › 2.Alpha_Diversity/alpha_rarefaction_plot/C372089/chao1.pdf]

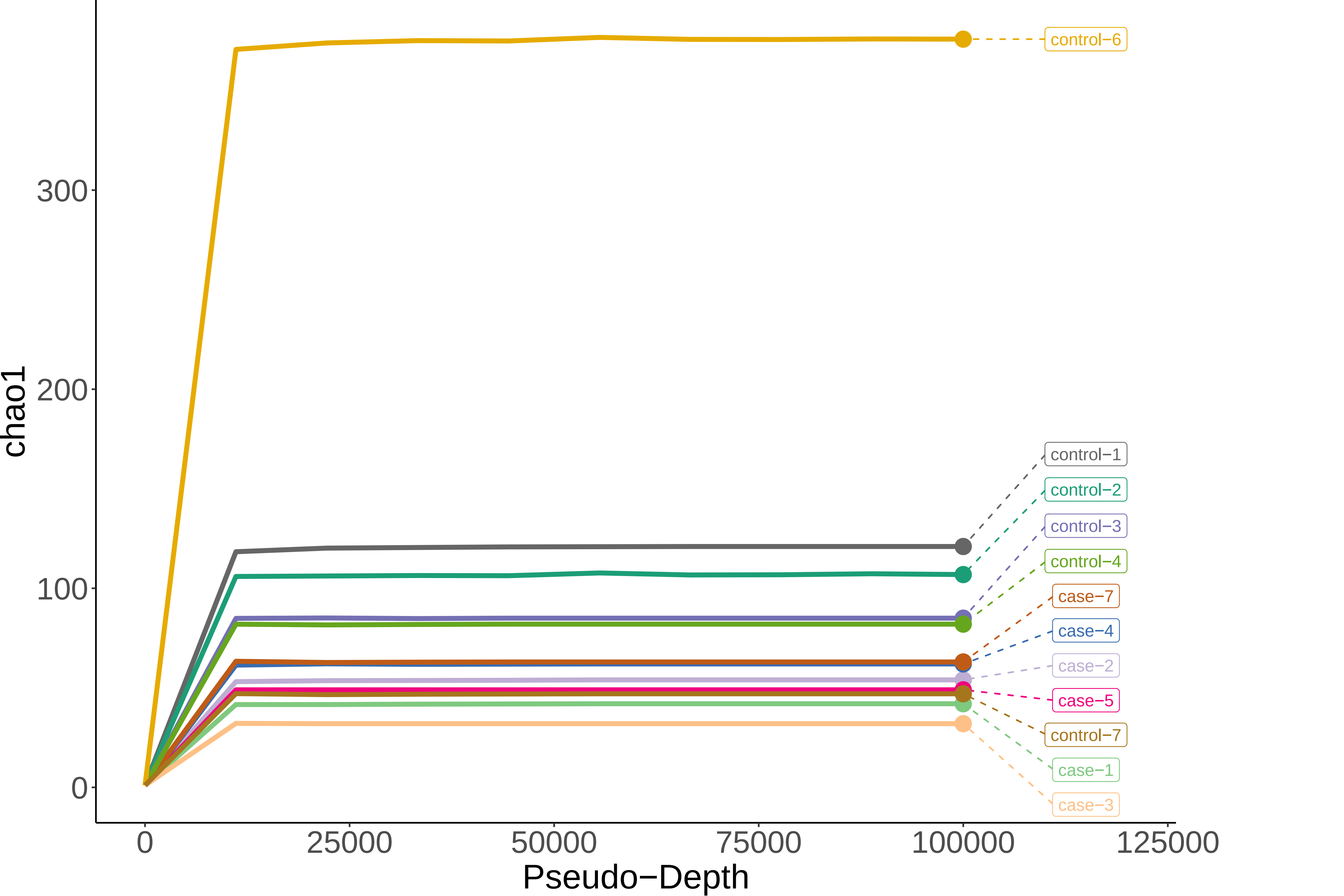
chao1
300
200
100
0
0
25000
50000
75000
100000
125000
Pseudo−Depth
control−6
control−1
control−2
control−3
control−4
case−7
case−4
case−2
case−5
control−7
case−1
case−3
